# p18/Lamtor1-mTORC1 Signaling Controls Development of Mucin-producing Goblet Cells in the Intestine

**DOI:** 10.1247/csf.20018

**Published:** 2020-07-08

**Authors:** Shizuka Ito, Shigeyuki Nada, Daisuke Yamazaki, Tetsuya Kimura, Kentaro Kajiwara, Hiroaki Miki, Masato Okada

**Affiliations:** 1 Department of Oncogene Research, Research Institute for Microbial Diseases, Osaka University, 3-1 Yamadaoka, Suita, Osaka 565-0871, Japan; 2 Department of Cellular Regulation, Research Institute for Microbial Diseases, Osaka University, 3-1 Yamadaoka, Suita, Osaka 565-0871, Japan

**Keywords:** mTORC1, p18/lamtor1, intestinal epithelium, goblet cells, mucin

## Abstract

Mechanistic target of rapamycin complex 1 (mTORC1) plays a pivotal role in controlling cell growth and metabolism in response to nutrients and growth factors. The activity of mTORC1 is dually regulated by amino acids and growth factor signaling, and amino acid-dependent mTORC1 activity is regulated by mTORC1 interaction with the Ragulator-Rag GTPase complex, which is localized to the surface of lysosomes via a membrane-anchored protein, p18/Lamtor1. However, the physiological function of p18-Ragulator-dependent mTORC1 signaling remains elusive. The present study evaluated the function of p18-mediated mTORC1 signaling in the intestinal epithelia using *p18* conditional knockout mice. In *p18* knockout colonic crypts, mTORC1 was delocalized from lysosomes, and *in vivo* mTORC1 activity was markedly decreased. Histologically, *p18* knockout crypts exhibited significantly increased proliferating cells and dramatically decreased mucin-producing goblet cells, while overall crypt architecture and enteroendocrine cell differentiation were unaffected. Furthermore, *p18* knockout crypts normally expressed transcription factors implicated in crypt differentiation, such as Cdx2 and Klf4, indicating that p18 ablation did not affect the genetic program of cell differentiation. Analysis of colon crypt organoid cultures revealed that both p18 ablation and rapamycin treatment robustly suppressed development of mucin-producing goblet cells. Hence, p18-mediated mTORC1 signaling could promote the anabolic metabolism required for robust mucin production in goblet cells to protect the intestinal epithelia from various external stressors.

## Introduction

Mechanistic target of rapamycin complex 1 (mTORC1) plays central roles in integrating growth factor and nutrient signals, controlling cell growth, metabolism, and homeostasis (Laplante and Sabatini, 2009; Liu and Sabatini, 2020). mTORC1 is composed of mTOR, Raptor, Deptor, PRAS40 and mLST8, and is activated in response to amino acids, insulin and ATP (Bar-Peled and Sabatini, 2014; Dibble and Manning, 2013; Laplante and Sabatini, 2013). mTOR is a serine/threonine kinase belonging to the PI3K-related kinase family and phosphorylates downstream targets such as S6 kinase (S6K), 4EBP1, TFEB, and ULK1, to promote protein translation, lysosome biogenesis, and lipid synthesis, and suppress autophagy (Dunlop and Tee, 2009; Neufeld, 2010). Abnormal mTORC1 signaling is associated with various human diseases such as cancer, diabetes, and neurological disorders (Hoeffer and Klann, 2010; Saxton and Sabatini, 2017), and hence the mTORC1 signaling pathway is considered a potential therapeutic target for these diseases (Ilagan and Manning, 2016).

mTORC1 is present in the cytoplasm under starvation conditions, but is activated at the surface of lysosomes in response to amino acids (Sancak *et al.*, 2008). Amino acids are sensed by specific sensor proteins, such as Sestrin2, leading to activation of the Ragulator-Rag GTPase complex, which is anchored to lysosomes, via the GTPase activating proteins Gator1 and Folliculin. The activated Ragulator-Rag GTPase complex then recruits mTORC1 to the surfaces of lysosomes (Dibble and Cantley, 2015; Sancak *et al.*, 2010). On the lysosomal membrane, Rheb GTPase directly activates mTORC1 in response to growth factor signals via inactivation of TSC1/2, a GTPase-activating protein complex of Rheb.

The lysosomal membrane-anchored protein p18/Lamtor1 is a core component of the Ragulator-Rag GTPase complex (Sancak *et al.*, 2010), and is required for its assembly, and for its anchoring to lysosomes (Yonehara *et al.*, 2017). p18 is ubiquitously expressed, and p18 ablation in mice causes an embryonic lethal phenotype accompanied by functional defects in endosome/lysosome organization and membrane protein transport (Nada *et al.*, 2009). p18-deficient cells exhibit substantial decreases in phosphorylation of the mTORC1 substrates, S6K and 4EBP1 (Soma-Nagae *et al.*, 2013; Yonehara *et al.*, 2017), indicating that p18 is required for activation of mTORC1 on lysosomes. Recent genetic studies further revealed that p18 plays crucial roles in epidermal development (Soma-Nagae *et al.*, 2013), polarization of M2 macrophages (Kimura *et al.*, 2016), T cell proliferation and function (Hosokawa *et al.*, 2017), and innate immune responses (Hayama *et al.*, 2018). However, its physiological functions, particularly in epithelial tissues, from which over 80% of human cancers develop, remain elusive.

Severe diseases such as colorectal cancer (CRC) and inflammatory bowel disease (IBD) develop in the intestinal epithelia. Upregulation of mTORC1 is associated with poor prognosis in CRC patients, and mTORC1 is deregulated in IBD (Francipane and Lagasse, 2014; Gulhati *et al.*, 2009; Zhang *et al.*, 2009). mTORC1 inactivation suppresses growth of APC-deficient adenomas due to inhibition of translational elongation (Faller *et al.*, 2015). Therefore, mTORC1 inhibitors have been considered as potential CRC therapeutics drugs, but the responses of solid tumors to mTORC1 inhibitors are modest (Liko and Hall, 2015; Wang and Zhang, 2014). Elucidating the roles of mTORC1 signaling in intestinal disorders is necessary for development of more targeted approaches to treat for intestinal diseases.

**Fig. 1:**
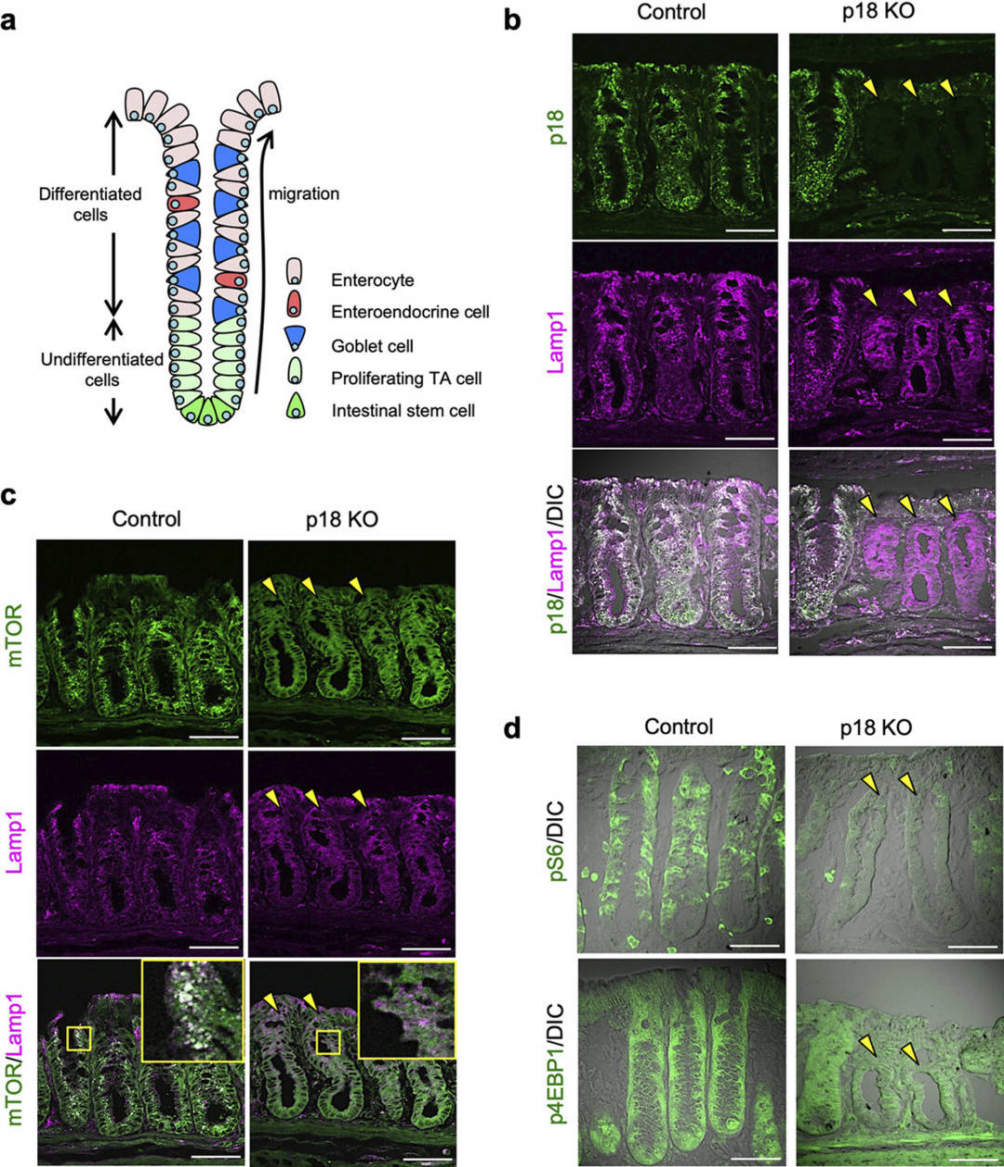
p18 ablation inactivated colon epithelium mTORC1 signaling. (a) Diagram of a normal colon crypt. Intestinal stem cells at the crypt base generate daily proliferating TA cells in the lower part of the crypt. TA cells differentiate into mature intestinal epithelial cells, including enterocytes, enteroendocrine cells and goblet cells, as the cells migrate to the top of crypt. (b) Immunofluorescence staining for p18 and Lamp1 in the colon epithelium of control and p18 KO mice. Differential interference contrast (DIC) images are also shown. Yellow arrowheads indicate p18 KO crypts. Scale bar, 50 μm. (c) p18 ablation decreased colon epithelium mTORC1 activity. Immunofluorescence staining of the mouse colon epithelium. Yellow arrowheads indicate p18 KO crypts. (d) Immunofluorescence staining for phospho-S6 (pS6) and phospho-4EBP1 (p4EBP1). DIC images are also shown. Yellow arrowheads indicate p18 KO crypts. Scale bar, 50 μm.

Intestinal epithelia undergo rapid turnover as a result of self-renewal and intestinal stem cell (ISC) proliferation (Barker, 2014) (Fig. 1a). mTORC1 regulates intestinal regeneration after injury (Sampson *et al.*, 2016), and a reduction in mTORC1 activity in Paneth cells, a key component of the ISC niche, promotes ISC proliferation (Yilmaz *et al.*, 2012). These findings suggest that mTORC1 signaling has a role in regulating the turnover and maintenance of intestinal tissues. However, neither the mechanisms governing ISC niche formation in colon epithelia lacking Paneth cells nor the mechanisms controlling proliferation/differentiation switching of intestinal epithelial cells are completely understood. Particularly, the roles of mTORC1 signaling regulated by the p18-Ragulator-Rag GTPase complex in intestinal epithelia have yet to be defined.

In the present study, we addressed the physiological function of p18-mediated mTORC1 signaling in the intestinal epithelium using *p18* conditional knockout mice. Histological analysis and *in vitro* analysis of organoid cultures revealed that the p18-mediated mTORC1 pathway was essential for the development of goblet cell functions, particularly the production of mucin that protects intestinal tissues from microbes, colitis, and cancer.

## Materials and Methods

### Antibodies

The following primary antibodies were used at the indicated dilutions: anti-Lamtor1 (p18) (Cell Signaling Technology, 8975, 1:500); anti-LAMP1 (Santa Cruz Biotechnology, sc-19992, 1:1000); anti-mTOR (Cell Signaling Technology, 2983, 1:500); anti-pS6 (Cell Signaling Technology, 4858, 1:500); anti-p4EBP1 (Cell Signaling Technology, 2855, 1:1000); anti-β-catenin (Cell Signaling Technology, 8480, 1:500); anti-E-cadherin (BD Biosciences, 610181, 1:200); anti-Ki67 (Thermo Fisher Scientific, 14-5698-82, 1:1000); anti-PCNA (Oncogene Science, NA03, 1:500); anti-Chromogranin A (Abcam, ab15160, 1:1000); anti-KLF4 (R&D systems, AF3158, 1:600); anti-CDX2 (Abcam, ab76541, 1:1000). The following secondary antibodies were used: Alexa Fluor 488-conjugated anti-rabbit, anti-mouse and anti-goat-IgG antibodies (Thermo Fisher Scientific, 1:1000) and Alexa Fluor 594-conjugated anti-rat-IgG antibody (Thermo Fisher Scientific, 1:1000).

### Mice

Generation and validation of *p18^flox/flox^* mice was described previously (Soma-Nagae *et al.*, 2013). *Ck19-CreErt2* transgenic mice (Means *et al.*, 2008) were crossed with *p18^flox/flox^* mice. F2 offspring carrying the *p18^flox/flox^* locus and the *Ck19-CreErt2* transgene, and their littermates carrying the *p18^flox/+^* locus and the *Ck19-CreErt2* transgene, were used as CK19-p18 KO mice and control mice, respectively. The following primers were used for genotyping:

p18-forward, 5'-AAGGATTCGGAGTTAGAGACTAGGAC-3';

p18-reverse, 5'-TGAGGATTCGAGTGGTGAGATACGA-3';

CK19-CreERT2-forward, 5'-AACATGCTTCATCGTCGGTCCGG-3';

CK19-CreERT2-reverse, 5'-CGGTATTGAAACTCCAGCGCGG-3'.

### Tamoxifen administration to mice

Tamoxifen solution was prepared as follows. Twenty mg of Tamoxifen (Sigma-Aldrich) was dissolved in 100 μL of ethanol using a homogenizer pestle, and then 900 μL of corn oil was added to the Tamoxifen/ethanol solution. Tamoxifen in this solution was completely dissolved at 37°C. Mice were administered 250 μg of 20 mg/mL Tamoxifen solution by oral gavage every other day for a total of three days. To increase the efficiency of Cre-loxP-mediated recombination in ISCs, mice were exposed to 3 Gy of X-ray irradiation after tamoxifen administration.

### Tissue staining

Four weeks after the first tamoxifen administration, mice were sacrificed using CO_2_ asphyxiation. Tissues were excised, cut open longitudinally on filter paper and washed with phosphate-buffered saline (PBS) to remove the contents. Tissues were fixed in 4% paraformaldehyde (PFA) in PBS for 2 h at 4°C and immersed sequentially in 10%, 20%, and 30% sucrose solutions. The tissues were then spooled around the toothpick, embedded in OCT compound (Sakura Finetek), and frozen on hexane cooled by liquid nitrogen. Frozen tissues were sliced into 6 μm sections using a cryostat (Leica). The sections were air-dried for 30 min and stored at –20°C. For immunofluorescence staining, frozen sections were immersed in HistoVT One (nacalai tesque, 1:10) for 20 min at 70°C for antigen activation, and permeabilized with 0.1% TritonX-100 in PBS for 30 min. After blocking with Blocking One (nacalai tesque) for 1 h, sections were incubated overnight at 4°C with primary antibodies in blocking solution (50% Can Get Signal, TOYOBO), followed by incubation with secondary antibodies for 1 h at room temperature. Cell nuclei were stained with 4',6'-diamidiono-2-phenylindole (DAPI). For hematoxylin and eosin staining (H&E), frozen sections were immersed in Mayer’s Hematoxylin Solution (Wako) for 4 min and washed with running water at 37°C for 10 min. After immersing with Eosin solution for 2.5 min, the sections were dehydrated with 70% ethanol and air-dried, followed by mounting with xylene and Entellan New (Merck Millipore). For alcian blue (AB) staining, frozen sections were immersed in 3% acetic acid for 3 min, and incubated in Alcian blue Solution pH 2.5 (Wako, 1:10) for 30 min. After immersing in 3% acetic acid for 3 min again, sections were washed with running water for 10 min. The sections were then stained with Hematoxylin Solution, dehydrated and mounted as described above.

### Transmission electron micrography

Tissue samples were fixed by cardiac perfusion with 2% formaldehyde and 2.5% glutaraldehyde in 0.1 M phosphate buffer (pH 7.4). Fixed tissues were cut into 2 mm slices and washed three times for 5 min each in 0.1 M phosphate buffer (pH 7.4) containing 4% sucrose. Tissues were post-fixed in 1% OsO_4_ and 0.5% potassium ferrocyanide in 0.1 M phosphate buffer (pH 7.4) for 60 min at room temperature. After washing in distilled water, samples were dehydrated in a graded series of ethanol solutions: 50%, 70%, and 90% ethanol on ice, and in 100% ethanol at room temperature for 10 min. Dehydrated samples were incubated twice in 100% propylene oxide (PO) for 5 min each, and then in 50% epoxy resin mixture dissolved in 100% propylene oxide (PO: resin=1:1) for 60 min more at room temperature. Sample tissues were incubated in pure epoxy resin mixture twice for 60 min each at room temperature, and embedded in epoxy resin for 2 days at 60°C. Eighty nm thick ultrathin sections were cut and stained with 2% uranyl acetate solution for 30 min, briefly washed three times in distilled water, and subsequently stained with lead staining solution for 2 min, and washed three times in distilled water. Electron micrographs were captured through a JEM 1400plus electron microscope (JEOL) at 80 kV with a VELETA CCD camera (2K×2K pixels, Olympus).

### Preparation of organoid cultures

Organoid culture conditioned medium was prepared as described in a previous report (Miyoshi and Stappenbeck, 2013) as follows. Primary culture medium was comprised of 20% (v/v) fetal bovine serum (FBS), penicillin/streptomycin and L-glutamine in Advanced Dulbecco’s Modified Eagle Medium/Ham’s F-12 (Invitrogen, 12634-010). L-WRN cells (ATCC, CRL-3276) were cultured in primary culture medium at 37°C in a humidified atmosphere containing 5% CO_2_ for 8 days, and the culture supernatant was then collected as conditioned medium daily. Medium diluted two-fold was used as 50% L-WRN medium, and medium diluted by 40-fold was used as 2.5% L-WRN medium. Colons were excised from mice and cut open longitudinally. Colons were minced into 5 mm pieces, and were then shaken in 20 mM EDTA in PBS for 2 h at 4°C. After removing the supernatant, the colon pieces were vigorously pipetted in 10% FBS in PBS to isolate colon crypts. The supernatant was passed through a 70 μm cell strainer and centrifuged at 2,500 rpm for 5 min. The pellets were embedded in Matrigel (Corning, 356231), and the culture plate was turned upside down after seeding in the center of each well of a 24-well plate. Matrigel was polymerized for 5–10 min at 37°C, and 500 μL of 50% L-WRN medium containing 10 μM Y-27632 (Cayman Chemical Company, 10005583) was added to the well. Medium was changed every 2 days.

### Organoid section staining

Cultured organoids were fixed in 4% PFA in PBS for 20 min at room temperature and washed with PBS. After incubation in 30% sucrose solution overnight, organoids were embedded in OCT compound and frozen. Frozen organoids were sectioned as were the frozen tissues. For immunofluorescence staining, frozen organoid sections were permeabilized with 0.03% TritonX-100 in PBS for 30 min. After blocking with Blocking One for 1 h, sections were incubated overnight at 4°C with primary antibodies in blocking solution, followed by incubation with secondary antibodies for 1 h at room temperature. In case of staining with AB solution subsequently, sections were fixed in 4% PFA in PBS for 10 min at room temperature. After washing with PBS, sections were stained with AB similarly to the tissue sections, but were instead incubated for 10 min.

## Results

### Mouse epithelial p18 ablation

To elucidate the physiological functions of p18-mediated mTORC1 signaling in the intestinal epithelia, we conditionally knocked out the *p18* gene in epithelial cells by crossing *p18^flox/flox^* mice (Soma-Nagae *et al.*, 2013) with cytokeratin 19 promoter-driven *CreErt2* (CK19-CreERT2) transgenic mice (Means *et al.*, 2008) (Fig. S1). Tamoxifen was administered intraperitoneally for 4 weeks to *p18^flox/flox^-Ck19-CreErt2* (CK19-p18 KO) mice, and colon tissues were collected. Longitudinal sections of colon tissues exhibiting multiple crypts (Fig. 1a) were analyzed by immunofluorescence for p18 and Lamp1, a lysosomal marker (Fig. 1b). In control tissues, p18 co-localized with lysosomes located on the apical sides of colon epithelial cells in the entire crypts (Fig. 1b, left panels). In the colons of CK19-p18 KO mice, however, p18 expression was entirely abolished in some crypts, while other crypts expressed p18 in most of the cells, similar to normal crypts (Fig. 1b, right panels).

p18 expression was also evaluated in the small intestine, which is composed of crypts and villi, by the same methods. As with the colon epithelia, p18 expression was entirely abolished in some crypts and villi in CK19-p18 KO mice, but other crypts and villi expressed p18 on lysosomes similarly to normal tissues (Fig. S2a–b). These results indicated that the *p18* gene was randomly deleted in the intestinal stem cells (ISCs) of colon and small intestinal crypts by *CK19-CreERT2*, likely due to limited activity of the CK19 promoter in the ISCs. This prompted us to evaluate the phenotypes of p18 KO crypts in comparison to p18-expressing crypts in the intestines of the same individual.

### p18 ablation attenuated colon epithelium mTORC1 activity

**Fig. 2:**
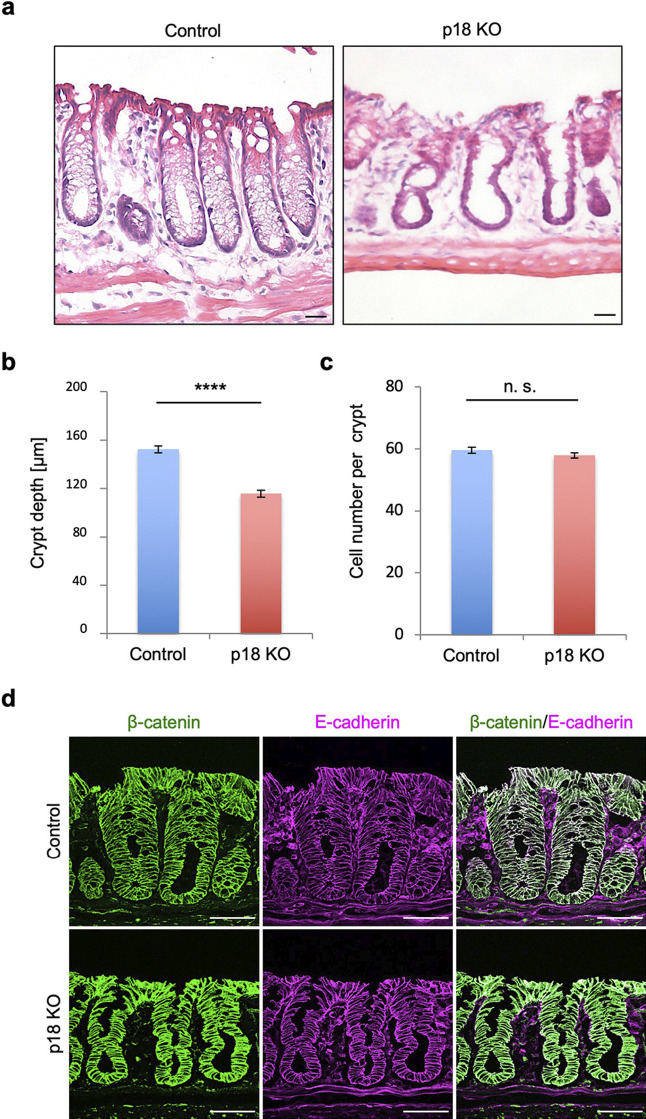
Effects of p18 ablation on crypt structure and expression of epithelial cell markers. (a) H&E staining of colon sections. Scale bar, 20 μm. (b) Quantification of crypt depth in control and p18 KO crypts. Values are representative of mean±s.e. n=44. ****p<0.0001, Student’s t-test. (c) Quantification of cell numbers per crypt in control and p18 KO crypts. Values are representative of mean±s.e. n=46. n.s., not significant, Student’s t-test. (d) Immunofluorescence staining of colon sections for epithelial cell markers β-catenin and E-cadherin. Scale bar, 50 μm.

A previous *in vitro* study revealed a crucial role for p18 in the regulation of mTORC1 activity (Sancak *et al.*, 2010; Yonehara *et al.*, 2017). To verify the role of p18 in the intestinal epithelium, mTORC1 activity was assessed in p18 KO crypts by immunofluorescence analysis. In control crypts, mTOR, a component of mTORC1, was localized to Lamp1-positive lysosomes (Fig. 1c, left panels). By contrast, mTOR was delocalized from lysosomes and diffused in the cytoplasm in p18 KO crypts (Fig. 1c, right panels). Consistent with the release of mTOR from lysosomes, phosphorylation of the representative substrates of mTORC1, S6 and 4EBP1, was markedly decreased in crypts exhibiting morphological features characteristic of p18 KO crypts as described later (Fig. 1d, Fig. 2a–b). These results demonstrated that mTORC1 activity was attenuated by p18 ablation in colon crypts.

### p18 ablation affected crypt structure and epithelial cell characteristics

Due to only partial p18 ablation in the colon epithelium of CK19-p18KO mice, mutant mice displayed no overt phenotype. Progeny were born healthy, grew normally, and did not exhibit apparent intestinal diseases. Thus, we examined the effects of p18 ablation on tissue architecture. Hematoxylin and eosin (H&E) staining revealed that mesh-like structures fully filled the luminal space of normal crypts, while in p18 KO crypts, the mesh-like structures were absent, and the luminal space was largely expanded (Fig. 2a). Furthermore, p18 KO crypts were shallower than control crypts (Fig. 2b). These characteristic features of p18 KO crypts allowed us to discriminate p18 KO crypts from control crypts even under DIC microscopy. However, there were no significant differences in colon epithelial cell numbers between control and p18 KO crypts (Fig. 2c). β-catenin and E-cadherin, which are epithelial cell markers, were expressed in p18 KO crypts similarly to control crypts, although they were more intensely concentrated in p18 KO crypts than in control crypts, potentially due to smaller cell sizes in p18 KO crypts (Fig. 2d). These data suggest that p18 ablation did not grossly affect the architecture of colon crypts, but induced significant structural and functional abnormalities in the colon epithelial cells.

### p18 ablation increased undifferentiated proliferating cells in the colon epithelium

**Fig. 3:**
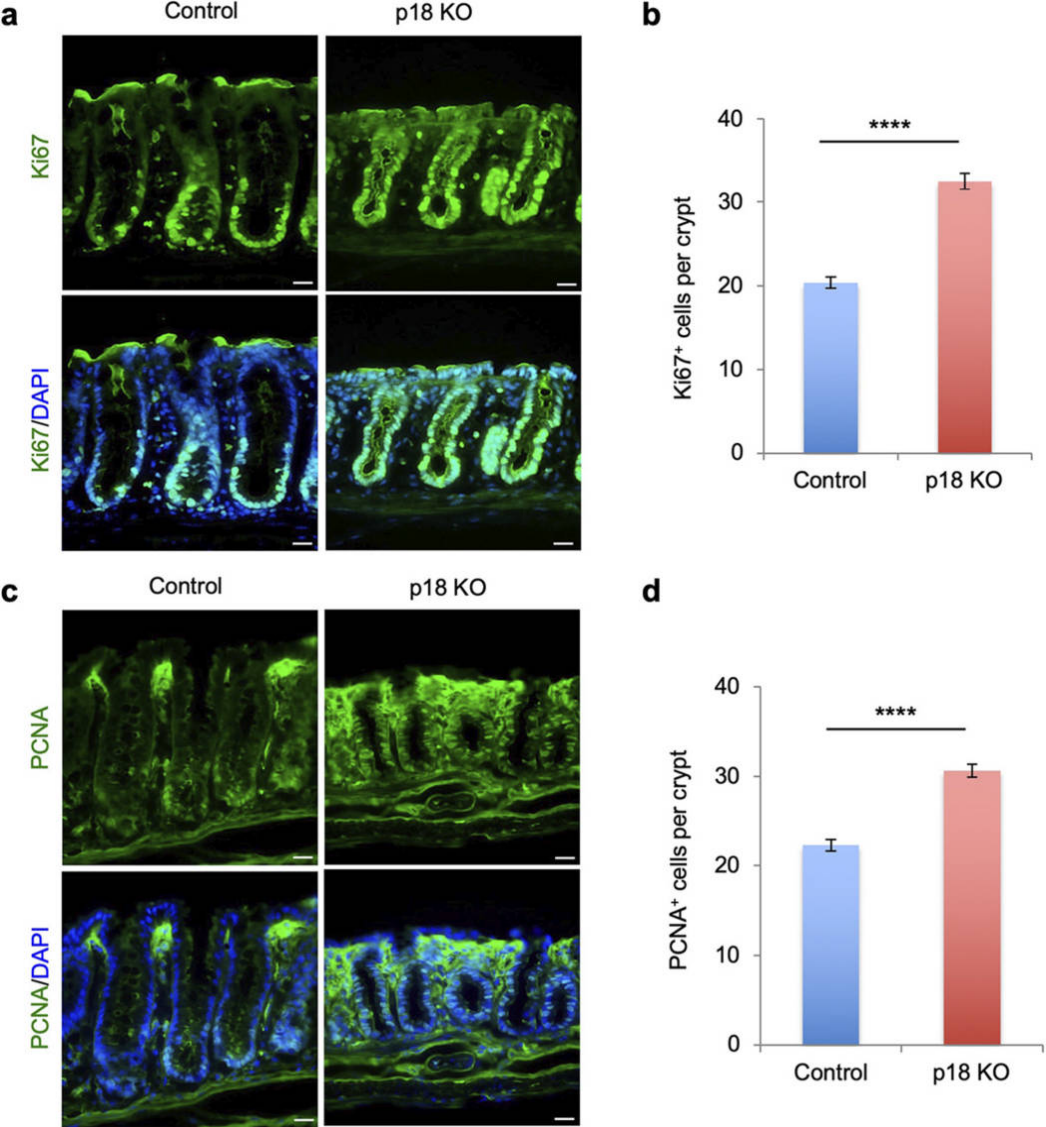
*p18* ablation increased undifferentiated proliferating cells in the colon epithelium. (a) Immunofluorescence staining of colon sections for Ki67. Cell nuclei are also stained with DAPI. Scale bar, 20 μm. (b) Quantification of Ki67^+^ cells per crypt in control and p18 KO crypts. Values are representative of mean±s.e. n=47. ****p<0.0001, Student’s t-test. (c) Immunofluorescence staining of colonic sections for PCNA. Cell nuclei are stained with DAPI. Scale bar, 20 μm. (d) Quantification of PCNA^+^ cells per crypt in control or p18 KO crypts. n=43. Values are representative of mean±s.e. n=43. ****p<0.0001, Student’s t-test.

In colonic crypts, undifferentiated proliferating transit amplifying (TA) cells are located in the proximal region, and differentiated cells dynamically migrate toward the distal side of the crypt (Barker, 2014) (Fig. 1a). We thus examined the effects of p18 ablation on the proliferation and differentiation of the colon epithelial cells. Immunofluorescence staining of the cell proliferation marker Ki67 revealed that Ki67^+^ cells were present at the proximal region in control crypts, while in p18 KO crypts, Ki67^+^ cells were increased overall, and extended further distally (Fig. 3a–b). PCNA immunostaining exhibited a similar pattern to Ki67 immunostaining (Fig. 3c–d). These results indicate that undifferentiated and proliferating TA cells were increased in p18 KO colon crypts.

### p18 ablation suppressed intestinal epithelial goblet cell development

**Fig. 4:**
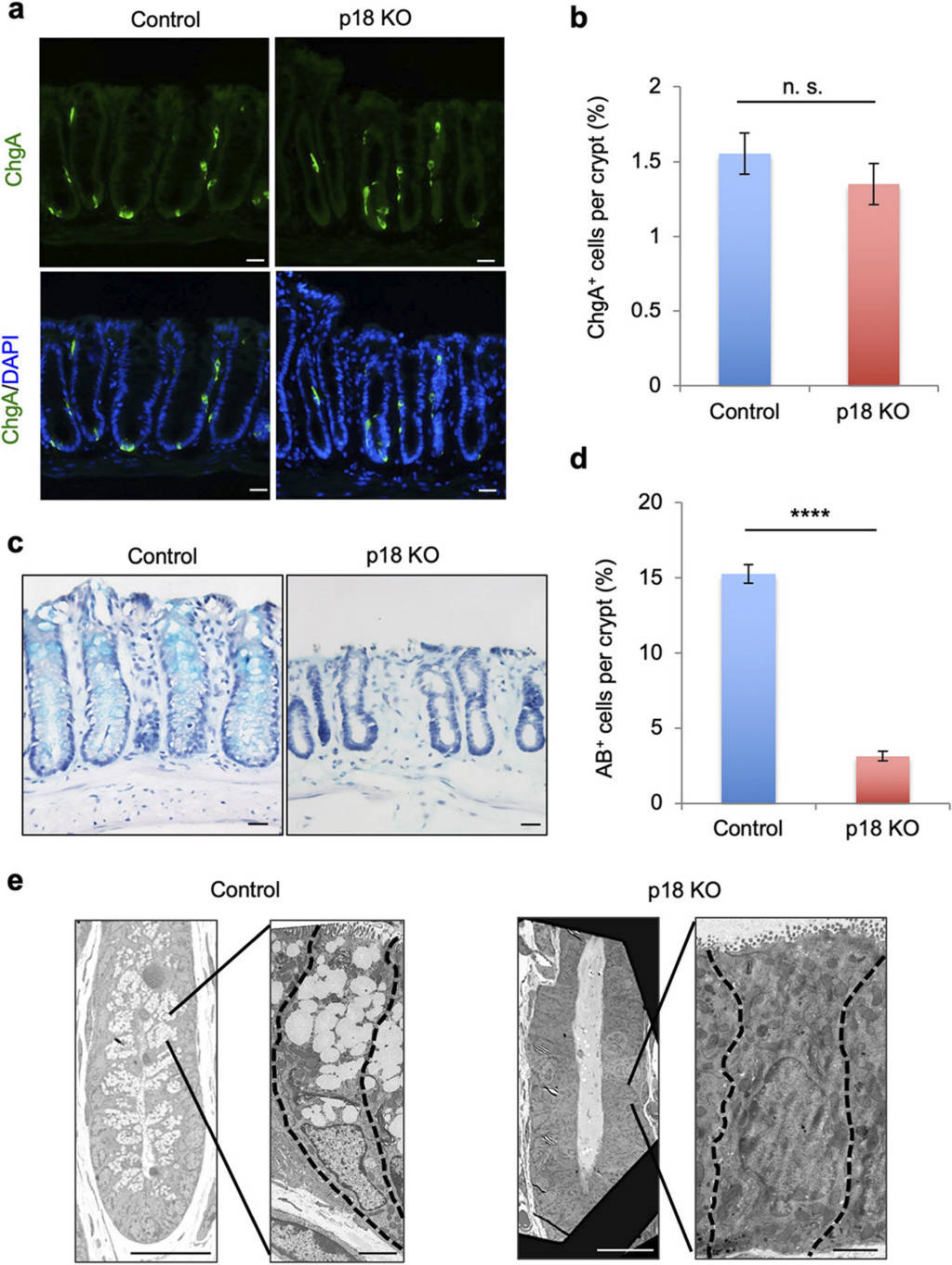
p18 ablation maintained enteroendocrine cells, but markedly decreased mucin-producing goblet cells in the colon epithelium. (a) Immunofluorescence staining for ChgA in colon sections. Cell nuclei are stained with DAPI. Scale bar, 20 μm. (b) Quantification of ChgA^+^ cells per crypt in control or p18 KO crypts. Values are representative of mean±s.e. n=74. n.s., not significant, Student’s t-test. (c) AB and hematoxylin staining of colon sections. Scale bar, 20 μm. (d) Quantification of AB^+^ cells per crypt in control and p18 KO crypts. Values are representative of mean±s.e. n=62. ****p<0.0001, Student’s t-test. (e) Representative transmission electron microscopy images of control and p18 KO crypts. Scale bars, 20 μm (left) and 2 μm (right).

Subsequently, we investigated whether p18 ablation affected differentiation of enteroendocrine cells and goblet cells, which are major differentiated colonic crypt cells. Immunofluorescence staining with the enteroendocrine cell marker Chromogranin A (ChgA) revealed no significant difference in ChgA^+^ cell numbers between control and p18 KO crypts (Fig. 4a–b). Contrastingly, staining with the goblet cell mucin marker Alcian blue (AB) revealed a dramatic decrease in AB^+^ cells in p18 KO crypts (Fig. 4c–d). Additionally, transmission electron microscopy revealed few or no mucin granules in p18 KO colon epithelial cells (Fig. 4e).

We also examined the effects of p18 ablation on the differentiation of small intestinal epithelial cells. In the small intestine, crypts are almost completely occupied by proliferating cells, which differentiate into functional cells in the villi (Barker, 2014). Ki67 immunostaining of the small intestine revealed unchanged abundance of Ki67^+^ cells between control and p18 KO crypts (Fig. S3a–b). However, the number of AB^+^ cells significantly decreased in p18 KO crypts, as in the colon epithelia (Fig. S4a–c). These results suggested that p18-mediated signaling could specifically regulate goblet cell differentiation or function, such as mucin production, in the intestinal tissues.

### p18 ablation did not affect Klf4 or Cdx2 expression

**Fig. 5:**
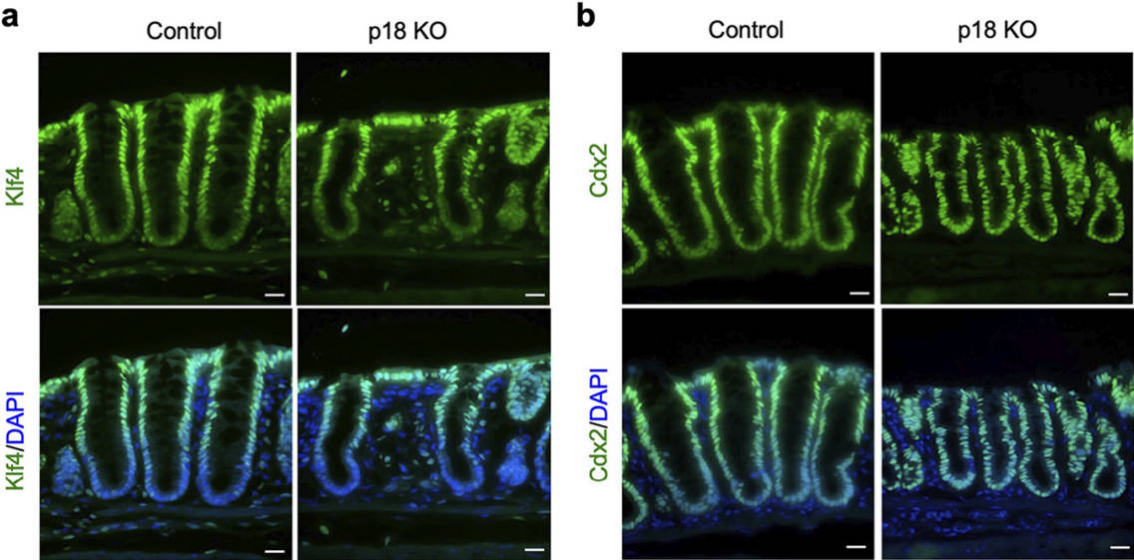
p18 ablation did not affect colon crypt expression Klf4 or Cdx2. (a) Immunofluorescence staining of colon sections for Klf4. Cell nuclei are stained with DAPI. Scale bar, 20 μm. (b) Immunofluorescence staining of colon sections for Cdx2. Cell nuclei are stained with DAPI. Scale bar, 20 μm.

To determine if p18-mediated signaling regulated cell differentiation or development of goblet cell functions, we measured expression of the transcription factors, Klf4 and Cdx2, in p18 KO crypts. Klf4 is involved in terminal goblet cell differentiation, and Cdx2 is a marker of the normal intestinal epithelial cells and regulates transcription of the mucin gene (Katz *et al.*, 2002; Saad, 2011). Immunofluorescence analysis revealed that Klf4 and Cdx2 were expressed in p18 KO crypts similarly to control crypts (Fig. 5a–b). These data suggested that p18 KO did not necessarily affect the genetic program of goblet cell differentiation, at least through these transcription factors.

### p18 ablation suppressed goblet cell development in organoid cultures

**Fig. 6:**
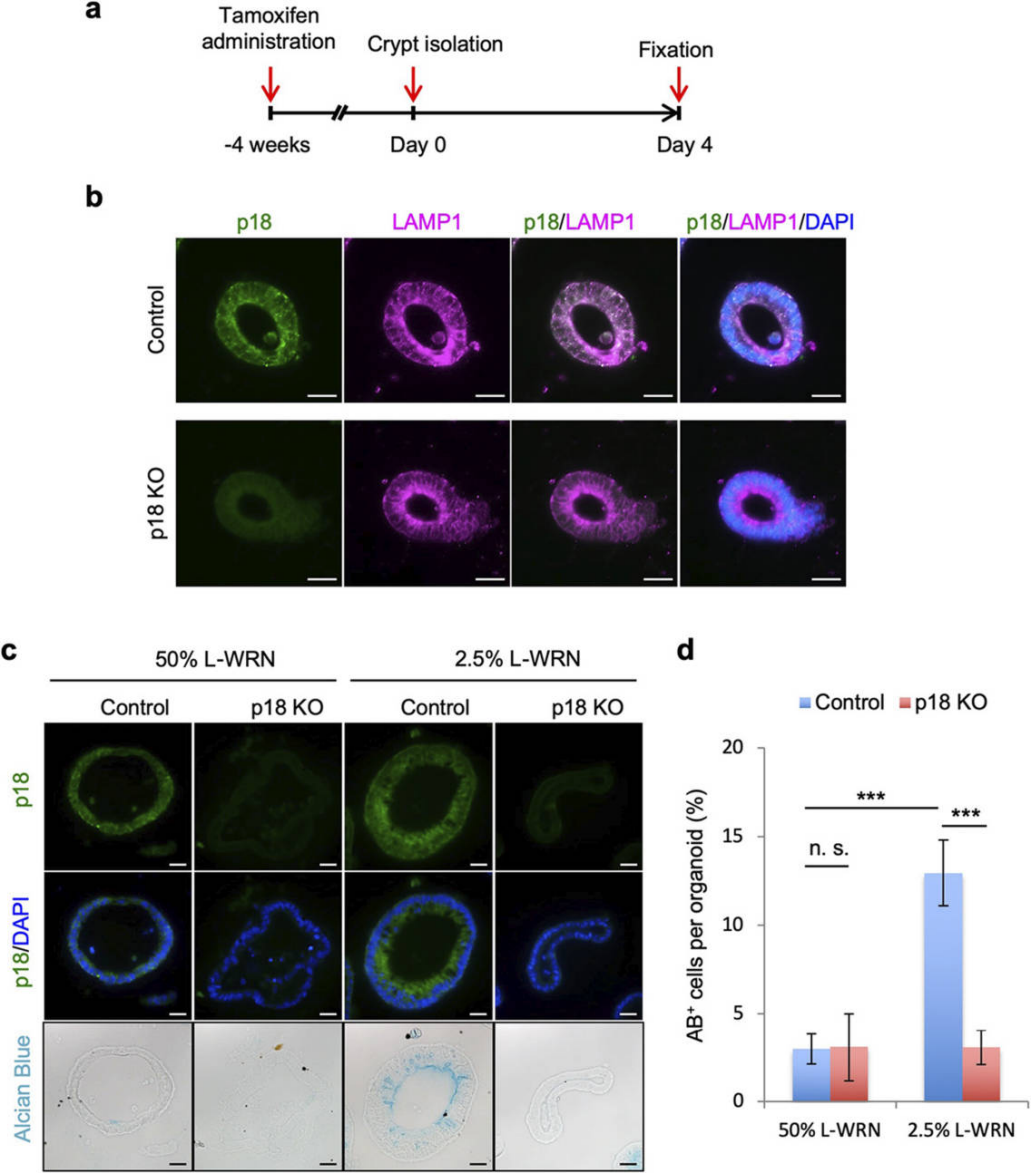
Mucin-producing goblet cells were decreased in p18 KO organoids. (a) Experimental scheme for colon organoid cultures. (b) Immunofluorescence staining of colon organoid sections for p18 and Lamp1. Cell nuclei are stained with DAPI. Scale bar, 20 μm. (c) Staining of colon organoid sections for p18, DAPI, and AB. After immunofluorescence staining, sections were stained with AB solution. Scale bar, 20 μm. (d) Quantification of AB^+^ cells per organoid. Values are representative of mean±s.e. ***p<0.001; n.s. not significant, Student’s t-test.

Tissue analysis suggested that p18 ablation suppressed development of goblet cell functions, including mucin production. To evaluate this possibility, we analyzed the *in vitro* differentiation of goblet cells using organoid cultures of colon crypts. Organoid culture is a three-dimensional *in vitro* culture system that exploits the self-organizing ability of ISCs allowing them to form a tissue-like anatomical structures. Based on a previous report (Miyoshi and Stappenbeck, 2013), we established an experimental system using organoid cultures derived from p18 KO colon epithelial cells (Fig. S5). Four days after starting organoid cultures, organoids were fixed, and frozen sections for immunostaining were prepared (Fig. 6a). Immunofluorescence analysis verified that both p18 KO and p18-expressing organoids were obtained from the colons of CK19-p18KO mice (Fig. 6b). These results indicated that p18 KO crypts were able to form organoids, and suggested that loss of p18 function did not affect the stemness of ISCs.

Using the above system, we assessed cell differentiation in p18 KO organoids. Intestinal organoids can ordinarily be cultured in growth medium containing abundant intestinal stem cell niche factors, such as Wnt3a, R-spondin3, and Noggin (WRN). These niche factors maintain cell proliferation and suppress cell differentiation, and organoid culture in medium lacking niche factors induces cell differentiation. We used niche factor-containing medium (50% L-WRN) to promote cell growth and niche factor-reduced medium (2.5% L-WRN) to induce differentiation. Two days after culture in 50% L-WRN, media was changed to 2.5% L-WRN in both treatment groups, and organoids were cultured for 2 days (Fig. 6c). AB staining revealed that mucin-producing goblet cells developed robustly in p18-expressing organoids, but were almost completely suppressed in p18 KO organoids (Fig. 6c–d). These findings demonstrated that p18 signaling was involved in the development of goblet cell functions *in vitro* as well as *in vivo*.

### p18 functioned via mTORC1 activation

**Fig. 7:**
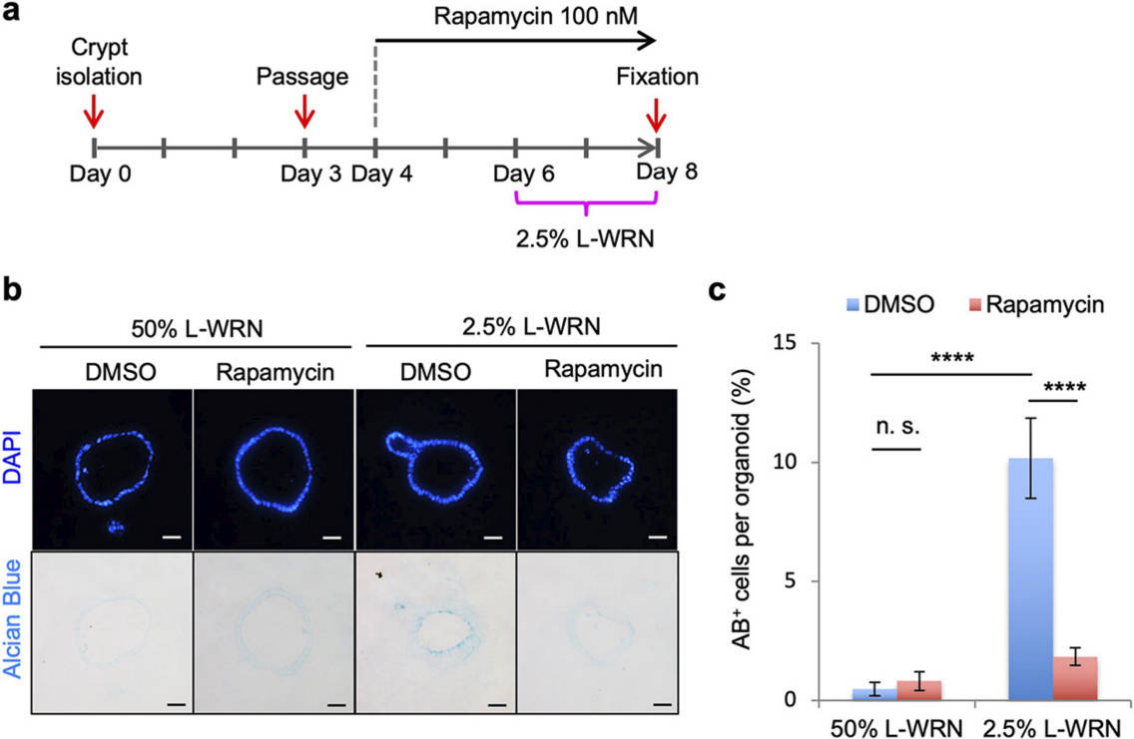
Effect of rapamycin treatment on the development of mucin-producing goblet cells in colon organoids. (a) Experimental schematic. (b) AB staining of colon organoid sections. Cell nuclei are stained with DAPI. Scale bar, 20 μm. (c) Quantification of the ratio of AB^+^ cells per organoid. The ratio was calculated from the number of AB^+^ cells per DAPI signal. Values are representative of mean±s.e. n=20-23. ****p<0.0001; n.s. not significant, Student’s t-test.

To determine if defective goblet cell function in p18 KO crypts and organoids was indeed mediated by mTORC1 signaling, we examined the effects of rapamycin-mediated inhibition of mTORC1 on the development of mucin-producing goblet cells in colon organoids. Control organoids cultured in 50% L-WRN were treated with or without rapamycin for 2 days, and differentiation was subsequently induced by changing the media to 2.5% L-WRN with or without rapamycin (Fig. 7a). AB staining revealed that rapamycin treatment robustly suppressed the development of mucin-producing goblet cells (Fig. 7b-c), as observed in p18 KO organoids (Fig. 6c–d). These observations, together with the finding that mTORC1 signaling was inactivated in p18 KO colon crypts, suggested that p18 plays crucial roles in the functional development of goblet cells via mTORC1 pathway activation.

## Discussion

In the present study, we analyzed the physiological functions of p18-mediated signaling in the colon and small intestine by generating CK19-p18 KO mice. Histological analysis revealed that p18 ablation markedly decreased the abundance of mucin-producing goblet cells in colon crypts, accompanied by downregulation of mTORC1 activity. However, the genetic program for goblet cell differentiation proceeded normally in p18 KO crypts. *In vitro* analysis of organoid cultures also revealed that p18 ablation and rapamycin-mediated inhibition of mTORC1 suppressed the development of mucin-producing goblet cells. These results suggested that the p18-mediated mTORC1 pathway is involved in the development of goblet cell functions, particularly mucin production. This mechanism could be crucial for protection of the intestinal epithelia from foreign substances, such as pathogenic microbes.

In the colon epithelium of CK19-p18 KO mice, we found that p18 KO crypts and p18-expressing crypts co-existed in the same colon tissues. ISCs residing at the crypt bottom generate proliferating TA cells daily, which differentiate into mature cells. The differentiated crypt cells turn over rapidly through apoptosis at the top of crypts (Barker *et al.*, 2007; Sato *et al.*, 2009). Thus, to generate p18 KO crypts, p18 needs to be deleted in ISCs by CK19-CreERT2. However, CK19 is not expressed in ISCs, but is highly expressed in the differentiated colon crypt epithelium (Brembeck *et al.*, 2001) and quiescent cells proximal to ISCs, referred to as “+4 cells” (Asfaha *et al.*, 2015). The +4 cells can dedifferentiate into ISCs when ISCs are injured by various stressors, such as irradiation (Barker *et al.*, 2012). We thus treated CK19-p18 KO mice with sublethal doses of X-ray irradiation in combination with tamoxifen administration. Although irradiated mice tended to exhibit larger regions of p18 KO crypts than non-irradiated mice, a substantial population of p18-expressing crypts remained. This implies that, even if p18 was ablated in some ISCs, p18 KO ISCs might need to occupy the entire ISC niche to generate p18 KO crypts. In this context, it is interesting to speculate that p18 KO ISCs could be eliminated via cell competition when normal ISCs coexist in the ISC niche or that a minor population of p18 KO ISCs generated from +4 cells could compete successfully to occupy the ISC niche.

We showed that p18 ablation suppressed the generation of mucin-expressing goblet cells, but the underlying mechanisms remained unclear. Considering the increase of proliferating cells in p18 KO crypts, p18 ablation could delay differentiation from proliferating TA cells to functional goblet cells. The Notch and Wnt signaling pathways play regulatory roles in cell proliferation and differentiation in the adult mammalian intestine (Korinek *et al.*, 1998; Sancho *et al.*, 2015; Sato *et al.*, 2011). TA cells differentiate into the two epithelial lineages, the absorptive lineage that produces enterocytes with robust Notch expression, and the secretory lineage that produces goblet and enteroendocrine cells with low Notch expression. We thus expected that the p18 ablation-induced aberrant activation of Notch signaling suppressed differentiation into goblet cells. However, this is unlikely, as enteroendocrine cells were not decreased in p18 KO crypts, and expression of the transcription factor Klf4, which is inhibited by Notch signaling (Zheng *et al.*, 2009), was unchanged. In addition, expression of the transcription factor Cdx2, which interacts with the *Muc2* promoter to activate its transcription (Yamamoto *et al.*, 2003), also remained unchanged in p18 KO crypts. Therefore, p18 ablation is unlikely to affect the genetic program of goblet cell differentiation.

We also observed that p18 ablation promoted cell proliferation, and did not affect the expression of basic cellular components such as E-cadherin and β-catenin, suggesting that p18 loss suppressed the specialized function of goblet cells, particularly the mass production of mucin proteins. Given that p18 ablation may not affect the genetic program of *Muc2* gene transcription, it is likely that the p18-mTORC1 pathway activates mucin protein synthesis by phosphorylating downstream targets, such as S6K and 4EBP1, which are involved in the promotion of ribosome biosynthesis, mRNA biogenesis, and Cap-dependent protein translation and elongation (Liu and Sabatini, 2020). The synthesized mucin is stored in mucin granules that occupy a large area of the goblet cell cytoplasm, resulting in a larger cell volume and a change in cell shape. Thus, the loss of mucin granules following p18 ablation might be attributable to morphological changes and a reduction in crypt depth in p18 KO crypts. Similar phenomena were previously observed when p18 was specifically ablated in the epidermis (Soma-Nagae *et al.*, 2013). p18 ablation severely suppressed the production of filaggrin, a major component of stratum corneum, and the accumulation of keratohyalin granules in the stratum granular cells, which leads to the loss of epidermal barrier function. Taken together, these findings suggest that the p18-mTORC1 pathway may widely contribute to the mass production of biomaterials required for the specialized function of terminally differentiated cells, such as those found in the epithelia and potentially the endocrine glands.

Mucins secreted by goblet cells play essential roles in intestinal homeostasis, forming a mucus layer to protect the intestinal epithelium from enteric bacteria (Okamoto and Watanabe, 2016). *Muc2* is the gene encoding a primary component of mucus, and Muc2-deficient mice develop colitis (Van der Sluis *et al.*, 2006). Ulcerative colitis (UC) and Crohn’s Disease are common inflammatory bowel diseases (IBDs). These diseases are considered incurable, and their regulatory mechanisms are not fully understood. However, goblet cells are decreased in the intestinal epithelia of UC patients (Gersemann *et al.*, 2009; Okamoto *et al.*, 2009), and mucins secreted by goblet cells suppress development of UC. Though inflammation was unaffected by partial intestinal p18 ablation in the present study, it is possible that more widespread intestinal p18 ablation could cause colitis. Furthermore, UC and Crohn’s disease patients are at increased risk of colorectal cancer (Axelrad *et al.*, 2016; Hernandez-Gea *et al.*, 2013). Considering this context, intestinal epithelium-specific p18 KO mice are a potential model system for pathological analysis of colitis and colitis-induced colorectal cancers.

## References

[B1] Asfaha, S., Hayakawa, Y., Muley, A., Stokes, S., Graham, T.A., Ericksen, R.E., Westphalen, C.B., Von Burstin, J., Mastracci, T.L., Worthley, D.L., Guha, C., Quante, M., Rustgi, A.K., and Wang, T.C. 2015. Krt19+/Lgr5- Cells Are Radioresistant Cancer-Initiating Stem Cells in the Colon and Intestine. Cell Stem Cell, 16: 627–638. 26046762 10.1016/j.stem.2015.04.013 PMC4457942

[B2] Axelrad, J.E., Lichtiger, S., and Yajnik, V. 2016. Inflammatory bowel disease and cancer: The role of inflammation, immunosuppression, and cancer treatment. World J. Gastroenterol., 22: 4794–4801. 27239106 10.3748/wjg.v22.i20.4794 PMC4873872

[B3] Bar-Peled, L. and Sabatini, D.M. 2014. Regulation of mTORC1 by amino acids. Trends Cell Biol., 24: 400–406. 24698685 10.1016/j.tcb.2014.03.003 PMC4074565

[B4] Barker, N. 2014. Adult intestinal stem cells: Critical drivers of epithelial homeostasis and regeneration. Nat. Rev. Mol. Cell Biol., 15: 19–33. 24326621 10.1038/nrm3721

[B5] Barker, N., Van Es, J.H., Kuipers, J., Kujala, P., Van Den Born, M., Cozijnsen, M., Haegebarth, A., Korving, J., Begthel, H., Peters, P.J., and Clevers, H. 2007. Identification of stem cells in small intestine and colon by marker gene Lgr5. Nature, 449: 1003–1007. 17934449 10.1038/nature06196

[B6] Barker, N., Van Oudenaarden, A., and Clevers, H. 2012. Identifying the stem cell of the intestinal crypt: Strategies and pitfalls. Cell Stem Cell, 11: 452–460. 23040474 10.1016/j.stem.2012.09.009

[B7] Brembeck, F.H., Moffett, J., Wang, T.C., and Rustgi, A.K. 2001. The keratin 19 promoter is potent for cell-specific targeting of genes in transgenic mice. Gastroenterology, 120: 1720–1728. 11375953 10.1053/gast.2001.24846

[B8] Dibble, C.C. and Manning, B.D. 2013. Signal integration by mTORC1 coordinates nutrient input with biosynthetic output. Nat. Cell Biol., 15: 555–564. 23728461 10.1038/ncb2763 PMC3743096

[B9] Dibble, C.C. and Cantley, L.C. 2015. Regulation of mTORC1 by PI3K signaling. Trends Cell Biol., 25: 545–555. 26159692 10.1016/j.tcb.2015.06.002 PMC4734635

[B10] Dunlop, E.A. and Tee, A.R. 2009. Mammalian target of rapamycin complex 1: Signalling inputs, substrates and feedback mechanisms. Cell. Signal., 21: 827–835. 19166929 10.1016/j.cellsig.2009.01.012

[B11] Faller, W.J., Jackson, T.J., Knight, J.R.P., Ridgway, R.A., Jamieson, T., Karim, S.A., Jones, C., Radulescu, S., Huels, D.J., Myant, K.B., Dudek, K.M., Casey, H.A., Scopelliti, A., Cordero, J.B., Vidal, M., Pende, M., Ryazanov, A.G., Sonenberg, N., Meyuhas, O., Hall, M.N., Bushell, M., Willis, A.E., and Sansom, O.J. 2015. MTORC1-mediated translational elongation limits intestinal tumour initiation and growth. Nature, 517: 497–500. 25383520 10.1038/nature13896 PMC4304784

[B12] Francipane, M.G. and Lagasse, E. 2014. mTOR pathway in colorectal cancer: An update. Oncotarget, 5: 49–66. 24393708 10.18632/oncotarget.1548 PMC3960188

[B13] Gersemann, M., Becker, S., Kübler, I., Koslowski, M., Wang, G., Herrlinger, K.R., Griger, J., Fritz, P., Fellermann, K., Schwab, M., Wehkamp, J., and Stange, E.F. 2009. Differences in goblet cell differentiation between Crohn’s disease and ulcerative colitis. Differentiation, 77: 84–94. 19281767 10.1016/j.diff.2008.09.008

[B14] Gulhati, P., Cai, Q., Li, J., Liu, J., Rychahou, P.G., Qiu, S., Lee, E.Y., Silva, S.R., Bowen, K.A., Gao, T., and Evers, B.M. 2009. Targeted inhibition of mammalian target of rapamycin signaling inhibits tumorigenesis of colorectal cancer. Clin. Cancer Res., 15: 7207–7216. 19934294 10.1158/1078-0432.CCR-09-1249 PMC2898570

[B15] Hayama, Y., Kimura, T., Takeda, Y., Nada, S., Koyama, S., Takamatsu, H., Kang, S., Ito, D., Maeda, Y., Nishide, M., Nojima, S., Sarashina-Kida, H., Hosokawa, T., Kinehara, Y., Kato, Y., Nakatani, T., Nakanishi, Y., Tsuda, T., Koba, T., Okada, M., and Kumanogoh, A. 2018. Lysosomal Protein Lamtor1 Controls Innate Immune Responses via Nuclear Translocation of Transcription Factor EB. J. Immunol., 200: 3790–3800. 29686050 10.4049/jimmunol.1701283

[B16] Hernandez-Gea, V., Toffanin, S., Friedman, S.L., and Llovet, J.M. 2013. Role of the microenvironment in the pathogenesis and treatment of hepatocellular carcinoma. Gastroenterology, 144: 512–527. 23313965 10.1053/j.gastro.2013.01.002 PMC3578068

[B17] Hoeffer, C.A. and Klann, E. 2010. mTOR signaling: At the crossroads of plasticity, memory and disease. Trends Neurosci., 33: 67–75. 19963289 10.1016/j.tins.2009.11.003 PMC2821969

[B18] Hosokawa, T., Kimura, T., Nada, S., Okuno, T., Ito, D., Kang, S., Nojima, S., Yamashita, K., Nakatani, T., Hayama, Y., Kato, Y., Kinehara, Y., Nishide, M., Mikami, N., Koyama, S., Takamatsu, H., Okuzaki, D., Ohkura, N., Sakaguchi, S., Okada, M., and Kumanogoh, A. 2017. Lamtor1 Is Critically Required for CD4(+) T Cell Proliferation and Regulatory T Cell Suppressive Function. J. Immunol., 199: 2008–2019. 28768723 10.4049/jimmunol.1700157

[B19] Ilagan, E. and Manning, B.D. 2016. Emerging role of mTOR in the response to cancer therapeutics. Trends Cancer, 2: 241–251. 27668290 10.1016/j.trecan.2016.03.008 PMC5033243

[B20] Katz, J.P., Perreault, N., Goldstein, B.G., Lee, C.S., Labosky, P.A., Yang, V.W., and Kaestner, K.H. 2002. The zinc-finger transcription factor Klf4 is required for terminal differentiation of goblet cells in the colon. Development, 129: 2619–2628. 12015290 10.1242/dev.129.11.2619 PMC2225535

[B21] Kimura, T., Nada, S., Takegahara, N., Okuno, T., Nojima, S., Kang, S., Ito, D., Morimoto, K., Hosokawa, T., Hayama, Y., Mitsui, Y., Sakurai, N., Sarashina-Kida, H., Nishide, M., Maeda, Y., Takamatsu, H., Okuzaki, D., Yamada, M., Okada, M., and Kumanogoh, A. 2016. Polarization of M2 macrophages requires Lamtor1 that integrates cytokine and amino-acid signals. Nat. Commun., 7: 13130. 27731330 10.1038/ncomms13130 PMC5064021

[B22] Korinek, V., Barker, N., Moerer, P., Van Donselaar, E., Huls, G., Peters, P.J., and Clevers, H. 1998. Depletion of epithelial stem-cell compartments in the small intestine of mice lacking Tcf-4. Nat. Genet., 19: 379–383. 9697701 10.1038/1270

[B23] Laplante, M. and Sabatini, D.M. 2009. mTOR signaling at a glance. J. Cell Sci., 122: 3589–3594. 19812304 10.1242/jcs.051011 PMC2758797

[B24] Laplante, M. and Sabatini, D.M. 2013. Regulation of mTORC1 and its impact on gene expression at a glance. J. Cell Sci., 126: 1713–1719. 23641065 10.1242/jcs.125773 PMC3678406

[B25] Liko, D. and Hall, M.N. 2015. mTOR in health and in sickness. J. Mol. Med., 93: 1061–1073. 26391637 10.1007/s00109-015-1326-7

[B26] Liu, G.Y. and Sabatini, D.M. 2020. mTOR at the nexus of nutrition, growth, ageing and disease. Nat. Rev. Mol. Cell Biol., 21: 183–203. 31937935 10.1038/s41580-019-0199-y PMC7102936

[B27] Means, A.L., Xu, Y., Zhao, A., Ray, K.C., and Gu, G. 2008. A CK19CreERT knockin mouse line allows for conditional DNA recombination in epithelial cells in multiple endodermal organs. Genesis, 46: 318–323. 18543299 10.1002/dvg.20397 PMC3735352

[B28] Miyoshi, H. and Stappenbeck, T.S. 2013. In vitro expansion and genetic modification of gastrointestinal stem cells in spheroid culture. Nat. Protoc., 8: 2471–2482. 24232249 10.1038/nprot.2013.153 PMC3969856

[B29] Nada, S., Hondo, A., Kasai, A., Koike, M., Saito, K., Uchiyama, Y., and Okada, M. 2009. The novel lipid raft adaptor p18 controls endosome dynamics by anchoring the MEK-ERK pathway to late endosomes. EMBO J., 28: 477–489. 19177150 10.1038/emboj.2008.308 PMC2657578

[B30] Neufeld, T.P. 2010. TOR-dependent control of autophagy: Biting the hand that feeds. Curr. Opin. Cell Biol., 22: 157–168. 20006481 10.1016/j.ceb.2009.11.005 PMC2854204

[B31] Okamoto, R., Tsuchiya, K., Nemoto, Y., Akiyama, J., Nakamura, T., Kanai, T., and Watanabe, M. 2009. Requirement of notch activation during regeneration of the intestinal epithelia. Am. J. Physiol. Gastrointest. Liver Physiol., 296: 1–5. 10.1152/ajpgi.90225.2008 19023031

[B32] Okamoto, R. and Watanabe, M. 2016. Role of epithelial cells in the pathogenesis and treatment of inflammatory bowel disease. J. Gastroenterol., 51: 11–21. 26138071 10.1007/s00535-015-1098-4

[B33] Saad, R.S., Ghorab, Z., Khalifa, M.A., and Xu, M. 2011. CDX2 as a marker for intestinal differentiation: Its utility and limitations. World J. Gastrointest. Surg., 3: 159–159. 22180832 10.4240/wjgs.v3.i11.159 PMC3240675

[B34] Sampson, L.L., Davis, A.K., Grogg, M.W., and Zheng, Y. 2016. MTOR disruption causes intestinal epithelial cell defects and intestinal atrophy postinjury in mice. FASEB J., 30: 1263–1275. 26631481 10.1096/fj.15-278606 PMC4750410

[B35] Sancak, Y., Peterson, T.R., Shaul, Y.D., Lindquist, R.A., Thoreen, C.C., Bar-Peled, L., and Sabatini, D.M. 2008. The Rag GTPases bind raptor and mediate amino acid signaling to mTORC1. Science, 320: 1496–1501. 18497260 10.1126/science.1157535 PMC2475333

[B36] Sancak, Y., Bar-Peled, L., Zoncu, R., Markhard, A.L., Nada, S., and Sabatini, D.M. 2010. Ragulator-Rag complex targets mTORC1 to the lysosomal surface and is necessary for its activation by amino acids. Cell, 141: 290–303. 20381137 10.1016/j.cell.2010.02.024 PMC3024592

[B37] Sancho, R., Cremona, C.A., and Behrens, A. 2015. Stem cell and progenitor fate in the mammalian intestine: Notch and lateral inhibition in homeostasis and disease. EMBO reports, 16: 571–581. 25855643 10.15252/embr.201540188 PMC4428041

[B38] Sato, T., Vries, R.G., Snippert, H.J., Van De Wetering, M., Barker, N., Stange, D.E., Van Es, J.H., Abo, A., Kujala, P., Peters, P.J., and Clevers, H. 2009. Single Lgr5 stem cells build crypt-villus structures in vitro without a mesenchymal niche. Nature, 459: 262–265. 19329995 10.1038/nature07935

[B39] Sato, T., Van Es, J.H., Snippert, H.J., Stange, D.E., Vries, R.G., Van Den Born, M., Barker, N., Shroyer, N.F., Van De Wetering, M., and Clevers, H. 2011. Paneth cells constitute the niche for Lgr5 stem cells in intestinal crypts. Nature, 469: 415–418. 21113151 10.1038/nature09637 PMC3547360

[B40] Saxton, R.A. and Sabatini, D.M. 2017. mTOR Signaling in Growth, Metabolism, and Disease. Cell, 168: 960–976. 28283069 10.1016/j.cell.2017.02.004 PMC5394987

[B41] Soma-Nagae, T., Nada, S., Kitagawa, M., Takahashi, Y., Mori, S., Oneyama, C., and Okada, M. 2013. The lysosomal signaling anchor p18/LAMTOR1 controls epidermal development by regulating lysosome-mediated catabolic processes. J. Cell Sci., 126: 3575–3584. 23781028 10.1242/jcs.121913

[B42] Van der Sluis, M., De Koning, B.A.E., De Bruijn, A.C.J.M., Velcich, A., Meijerink, J.P.P., Van Goudoever, J.B., Büller, H.A., Dekker, J., Van Seuningen, I., Renes, I.B., and Einerhand, A.W.C. 2006. Muc2-Deficient Mice Spontaneously Develop Colitis, Indicating That MUC2 Is Critical for Colonic Protection. Gastroenterology, 131: 117–129. 16831596 10.1053/j.gastro.2006.04.020

[B43] Wang, X.W. and Zhang, Y.J. 2014. Targeting mTOR network in colorectal cancer therapy. World J. Gastroenterol., 20: 4178–4188. 24764656 10.3748/wjg.v20.i15.4178 PMC3989954

[B44] Yamamoto, H., Bai, Y.Q., and Yuasa, Y. 2003. Homeodomain protein CDX2 regulates goblet-specific MUC2 gene expression. Biochem. Biophys. Res. Commun., 300: 813–818. 12559945 10.1016/s0006-291x(02)02935-2

[B45] Yilmaz, Ö.H., Katajisto, P., Lamming, D.W., Gültekin, Y., Bauer-Rowe, K.E., Sengupta, S., Birsoy, K., Dursun, A., Onur Yilmaz, V., Selig, M., Nielsen, G.P., Mino-Kenudson, M., Zukerberg, L.R., Bhan, A.K., Deshpande, V., and Sabatini, D.M. 2012. MTORC1 in the Paneth cell niche couples intestinal stem-cell function to calorie intake. Nature, 486: 490–495. 22722868 10.1038/nature11163 PMC3387287

[B46] Yonehara, R., Nada, S., Nakai, T., Nakai, M., Kitamura, A., Ogawa, A., Nakatsumi, H., Nakayama, K.I., Li, S., Standley, D.M., Yamashita, E., Nakagawa, A., and Okada, M. 2017. Structural basis for the assembly of the Ragulator-Rag GTPase complex. Nat. Commun., 8: 1625. 29158492 10.1038/s41467-017-01762-3 PMC5696360

[B47] Zhang, Y.J., Dai, Q., Sun, D.F., Xiong, H., Tian, X.Q., Gao, F.H., Xu, M.H., Chen, G.Q., Han, Z.G., and Fang, J.Y. 2009. mTOR signaling pathway is a target for the treatment of colorectal cancer. Ann. Surgical Oncol., 16: 2617–2628. 10.1245/s10434-009-0555-9 19517193

[B48] Zheng, H., Pritchard, D.M., Yang, X., Bennett, E., Liu, G., Liu, C., and Ai, W. 2009. KLF4 gene expression is inhibited by the notch signaling pathway that controls goblet cell differentiation in mouse gastrointestinal tract. Am. J. Physiol. Gastrointest. Liver Physiol., 296: 490–498. 10.1152/ajpgi.90393.2008 PMC2660173 19109406

