# Supplementary material for: p18/Lamtor1-mTORC1 Signaling Controls Development of Mucin-producing Goblet Cells in the Intestine
Source: Cell Struct Funct. 2020 Jul 8;45(2):93–105. doi: 10.1247/csf.20018 (PMC10511045; doi:10.1247/csf.20018)
Supplement: Supplementary file 1 — Fig. S1 [file csf_45_20018_1.pdf]

Supplementary figures

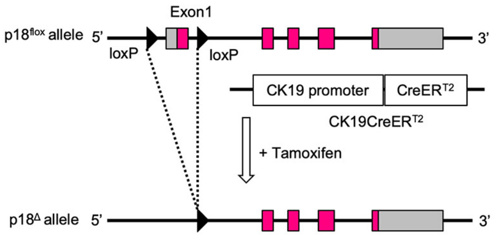

Figure S1: Conditional p18 knockout in the colon epithelium

Floxed and deleted p18 alleles and the CK19-CreERT2 transgene construct are shown.
