# Supplementary material for: p18/Lamtor1-mTORC1 Signaling Controls Development of Mucin-producing Goblet Cells in the Intestine
Source: Cell Struct Funct. 2020 Jul 8;45(2):93–105. doi: 10.1247/csf.20018 (PMC10511045; doi:10.1247/csf.20018)
Supplement: Supplementary file 2 — Fig. S2 [file csf_45_20018_2.pdf]

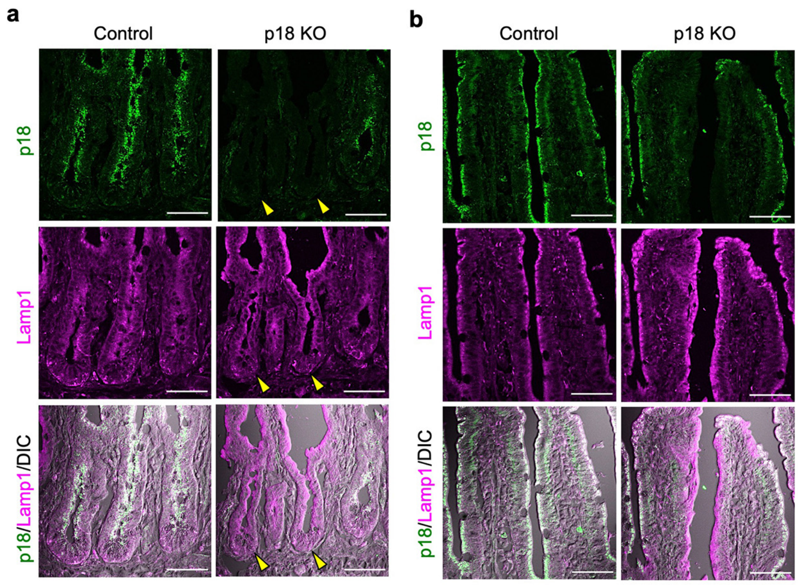

Figure S2: Small intestinal epithelium p18 knockout. Immunofluorescence staining for p18 and Lamp1 in the **(a)** crypts and **(b)** villi of small intestinal epithelium of control and p18 KO mice. DIC images are also shown. Yellow arrowheads indicate p18 KO crypts. Scale bar, 50 µm.
