# Supplementary material for: p18/Lamtor1-mTORC1 Signaling Controls Development of Mucin-producing Goblet Cells in the Intestine
Source: Cell Struct Funct. 2020 Jul 8;45(2):93–105. doi: 10.1247/csf.20018 (PMC10511045; doi:10.1247/csf.20018)
Supplement: Supplementary file 3 — Fig. S3 [file csf_45_20018_3.pdf]

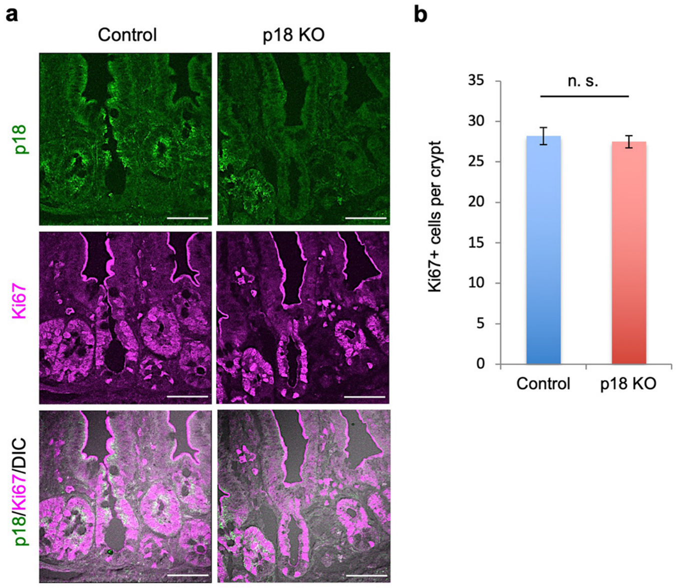


**Figure S3: p18 ablation did not affect cell proliferation in small intestinal epithelium crypts.** (**a**) Immunofluorescence staining for p18 and Ki67 in small intestine sections. DIC images are also shown. Scale bar, 50 μm. (**b**) Quantification of $Ki67^{+}$ cells per crypt in control and p18 KO crypts in the small intestine. Values are representative of mean ± s.e. control n = 30, p18 KO n = 27. n.s., not significant, Student's t-test.
