# Supplementary material for: p18/Lamtor1-mTORC1 Signaling Controls Development of Mucin-producing Goblet Cells in the Intestine
Source: Cell Struct Funct. 2020 Jul 8;45(2):93–105. doi: 10.1247/csf.20018 (PMC10511045; doi:10.1247/csf.20018)
Supplement: Supplementary file 4 — Fig. S4 [file csf_45_20018_4.pdf]

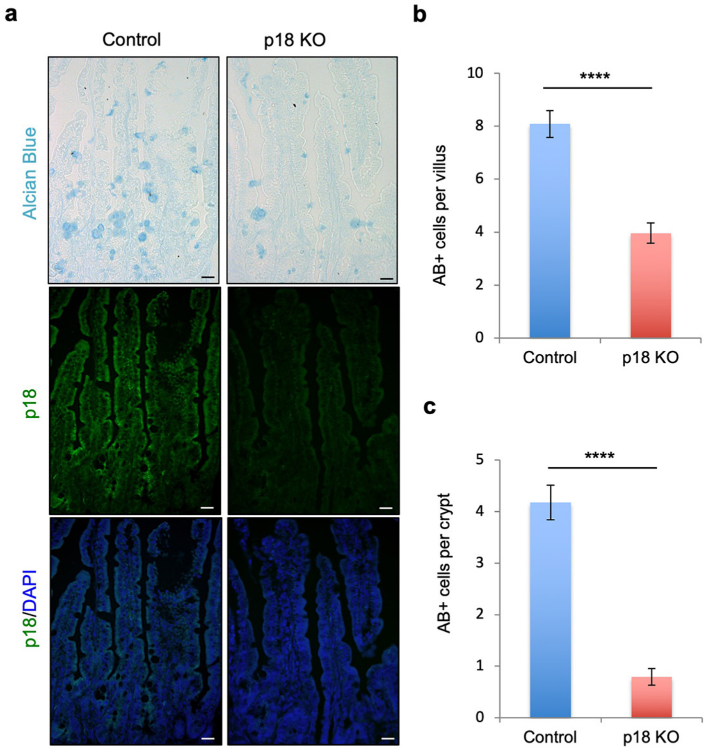

Figure S4: p18 ablation decreased small intestinal epithelial mucin-producing goblet cells. (**a**) AB staining and immunofluorescence for p18 in small intestine sections. Cell nuclei are stained with DAPI. scale bar, 20 µm. (**b**) Quantification of AB^+ cells per villus in control and p18 KO villi. Values are representative of mean ± s.e. n = 25. ****p < 0.0001, Student's t-test. (**c**) Quantification of AB^+ cells per crypt in control and p18 KO crypts. Values are representative of mean ± s.e. n = 34. ****p < 0.0001, Student's t-test.
