# Supplementary material for: p18/Lamtor1-mTORC1 Signaling Controls Development of Mucin-producing Goblet Cells in the Intestine
Source: Cell Struct Funct. 2020 Jul 8;45(2):93–105. doi: 10.1247/csf.20018 (PMC10511045; doi:10.1247/csf.20018)
Supplement: Supplementary file 5 — Fig. S5 [file csf_45_20018_5.pdf]

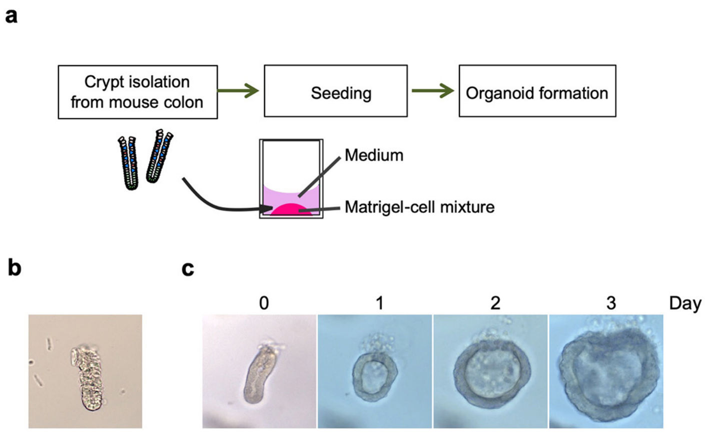

Figure S5: Formation of organoids from 18 KO crypts. (**a**) Flowchart of organoid culture. Four weeks after tamoxifen administration, colons were excised from mice and treated with EDTA to weaken crypt-to-tissue adhesion. Colon crypts were physically isolated from colon tissues by pipetting, and embedded in Matrigel, followed by incubation with 50% L-WRN medium. (**b**) A crypt isolated from colon tissue. (**c**) Day-by-day observation of a single crypt growing into spherical organoid.
